# Application of Continuous Culture Methods to Recombinant Protein Production in Microorganisms

**DOI:** 10.3390/microorganisms6030056

**Published:** 2018-06-21

**Authors:** Karl Peebo, Peter Neubauer

**Affiliations:** 1 Center of Food and Fermentation Technologies, Akadeemia tee 15a, 12618 Tallinn, Estonia; karl@tftak.eu; 2 Department of Chemistry and Biotechnology, Tallinn University of Technology, Akadeemia tee 15, 12618 Tallinn, Estonia; 3 Department of Bioprocess Engineering, Technische Universität Berlin, Ackerstraβe 76, ACK24, D-13355 Berlin, Germany

**Keywords:** continuous cultures, continuous manufacturing, recombinant protein production, steady state, specific growth rate, chemostat

## Abstract

Depending on the environmental conditions, cells adapt their metabolism and specific growth rate. Rearrangements occur on many different levels such as macromolecular composition, gene and protein expression, morphology and metabolic flux patterns. As the interplay of these processes also determines the output of a recombinant protein producing system, having control over specific growth rate of the culture is advantageous. Continuous culture methods were developed to grow cells in a constant environment and have been used for decades to study basic microbial physiology in a controlled and reproducible manner. Our review summarizes the uses of continuous cultures in cell physiology studies and process development, with a focus on recombinant protein-producing microorganisms.

## 1. Introduction

Efficient strategies to produce recombinant proteins are gaining increasing importance, as more applications that require high amounts of high-quality proteins reach the market [1]. Higher production efficiencies, faster process development and deeper understanding of basic cellular physiology are required to advance the field. As recombinant protein production is built upon living organisms, the process development is almost never straightforward and often demands an empirical approach. To manipulate and understand cells better, they should be grown in a defined, ideally constant, controllable set of physico-chemical conditions [2]. The most basic system that can provide such conditions is a chemostat where cells are maintained in a steady-state growth environment by supplying them with a constant flow of nutrients and by simultaneous removal of spent culture medium at a defined rate [3,4]. Other parameters, such as temperature, pH, and oxygenation rate can be varied and controlled by the experimenter. Several variations of the basic chemostat cultivation method have been developed to study cells more rapidly while keeping the steady-state environment (for a review of the methods see [5]). Most often, continuous cultures have been applied to basic physiology studies of wild-type cells; but as the final volumetric productivity of a protein production process depends both on the specific product formation rate (q_p_) and the specific growth rate (μ), methods that allow the experimenter to control and monitor μ can also be advantageous for process development purposes. Besides microbial physiology studies and process development, there is an interest in continuous cultures to manufacture recombinant proteins [6,7], as the possibility of keeping cells in producing states for longer times has the potential to significantly increase the process productivities and reduce costs [8,9].

In this review, we summarize how continuous cultures have been used to understand basics of recombinant cell physiology, for process development and industrial production of recombinant proteins. As semi-continuous perfusion processes have been used for mammalian cell-based recombinant processes for a long time and reviewed elsewhere [10,11,12], our review focuses on microorganisms.

## 2. Specific Growth Rate Dependent Metabolism of Microorganisms

Host cells can be considered as catalysts in the process of producing recombinant proteins. When a genetic element designed to produce a protein of interest is placed into host cells, its output depends strongly on the physiological state. One of the key characteristic of the physiological state of cells is their specific growth rate (µ) [13]. Most changes in physico-chemical growth environment will have an influence on the µ of cells. Additionally, for recombinant protein production it is common to use strong expression systems that can also significantly burden host cells and influence µ.

Schaechter et al. first showed that the macromolecular composition (contents of ribonucleic acid (RNA), DNA, and protein) of *Salmonella typhimurium* varies strongly according to how fast the cells are growing [14]. This has been shown to apply also to many other microorganisms [15,16,17]. In addition to macromolecular composition, all core parameters in the process of recombinant protein production-plasmid copy number [18], transcription [19], and translation rate [20,21] are changing with µ. Besides these general processes, recent genome-wide studies have shown that a large fraction of the transcriptome, proteome, and fluxome in microorganisms seem to be coordinated with µ [22,23,24,25]. Interestingly, the trends seem to be conserved between different organisms: commonly with faster growth increase in the concentration of essential and conserved gene products such as transcription and translation related proteins can be observed [22,26,27]. With this, downregulation of less-essential proteins with functions such as signaling, external stimuli sensing, proteolysis, and motility usually occurs. Similar trends can be observed even when changes in µ are caused by genome modifications, which complicates the selection of best producing strains in conditions where µ cannot be controlled (e.g., batch) [28]. Overflow metabolism is another example of µ-dependent rearrangement of metabolism [29,30] that has been linked to reduced recombinant protein production [31]. This is a phenomenon in which microorganisms growing faster than a certain threshold µ start using fermentation—a much less efficient way than aerobic respiration for generating energy even in the presence of oxygen.

Therefore, if processes are carried out in continuous cultures where µ can be fixed, the interpretation and control of processes can be simplified as metabolic rearrangements due to changing µ can be avoided.

## 3. μ-Dependent Recombinant Product Formation Kinetics

Chemostat cultures allow tight control over all growth conditions and the option to modify μ of host cells by limiting the feeding rate of an essential growth substrate. For this reason, chemostats have been widely used to determine the product formation kinetics—the relationship between µ and q_p_—in many different organisms. These process characteristics can be directly compared between different experimental setups as they are independent of process specific settings such as biomass density and reactor volume. We summarize continuous cultures studies reporting product formation kinetics in Table 1.

When comparing the maximal q_p_ achieved in different μ-dependent studies, the highest q_p_ values have been reached with *Escherichia coli.* With the strong inducible T7 promoter, as high as 75% of total protein production directed towards recombinant production has been reported [32]. Commonly with this promoter, growth coupled increase in q_p_ has been observed [32,33]. For other continuous processes with *E. coli*, bell-shaped kinetics with a maximum q_p_ most frequently achieved at medium µ values have been reported [34,35,36,37]. The decrease of q_p_ above a threshold µ often co-occurred with high acetate production, which probably limits the ability to reach a higher q_p_ with faster growth [35,36].

Probably the most thorough µ-dependent recombinant process analysis has been carried out using *Pichia pastoris,* recently summarized by Looser et al. [38]. In *P. pastoris*, most often two expression systems are used: (1) methanol inducible AOX1 (alcohol oxidase (1)) and (2) constitutive glycolytic GAP promoter (glyceraldehyde-3-phosphate dehydrogenase). For the methanol-inducible AOX expression system, bell-shaped protein formation kinetics with a maximum q_p_ near low growth rates (µ < 0.1 h^−1^) have most commonly been observed [38,39,40], although growth coupled kinetics have also been observed [41,42,43]. Possibly, these differences arise from the fact that methanol can been used both as an inducer or the carbon source by *P. pastoris*. Overall, a low µ range has been demonstrated to be optimal for AOX promoter-dependent protein expression. In *S. cerevisiae*, a galactose-dependent expression system that is induced by galactose and repressed by glucose is often used. With this promoter system used in galactose-based media, growth coupled q_p_ has been observed [44]. In contrast, glucose and galactose co-feeding resulted in a bell shaped q_p_ kinetics, possibly because of increasing inhibiting glucose concentrations at higher µ [45,46]. For the constitutive expression system relying on glycolytic GAP promoter in *P. pastoris*, a growth coupled increase of q_p_ with increasing μ has been observed [27,47,48,49]. For GAP promoter-based systems highest q_p_ values are commonly reported at the maximum µ values above 0.15 h^−1^. Similar growth coupled product formation kinetics have been reported for other yeast processes utilizing glycolytic promoters [50,51,52]. Transcriptome analysis of glycolytic promoter-dependent protein expression in *S. cerevisiae* [51] and *P. pastoris* [27] was used for a more detailed understanding of these processes. Both studies implied upregulation of stress response and endoplasmic reticulum functions, and downregulation of proteasome activities play key roles in the increased productivity with faster growth in these systems.

In contrast to most processes with yeast and bacteria, protein expression in *Aspergillus niger* has often been carried out using chromosomal expression instead of plasmid-based systems [53,54,55,56]. Often, hosts with multiple copies of the target gene are used for stronger expression [53,55]. In most cases, the product formation kinetics seem to be growth coupled and highest q_p_ have been reached at µ > 0.1 h^−1^ [53,54,55,56].

In summary, growth coupled product formation kinetics seems more common in continuous cultures. This is probably one of the reasons why often satisfactory yields are reached using simple batch cultures where µ is not restricted. Although empirical determination of optimal µ for each new recombinant process should be carried out, some general trends can be estimated beforehand depending on the host organism, promoter system and induction strategy.

## 4. Advanced Methods for μ-Dependent Recombinant Product Formation Kinetics

Applying chemostat cultivations for each new process development can be laborious and time consuming. Chemostat starts as a batch culture, after which the culture is typically stabilized for five bioreactor volume changes. This can take many days depending on the specified dilution rate [59]. To increase throughput of continuous cultures, parallelization and miniaturization have been used. For example, miniature chemostat systems have been developed for microbial physiology studies [24,60,61]. Recently they have also been applied to optimize induction conditions in parallel fermentations of recombinant protein expressing *E. coli* [62]. The miniature parallel chemostat was further advanced to a cascade chemostat system where one reactor was used for biomass production and the second reactor for the induction of recombinant protein expression [63]. Another multi-reactor design was demonstrated by Erm et al., who used a single chemostat reactor to seed multiple subsequent chemostats and study different recombinant expression induction strategies in *E. coli* [64]. These multistage cultivation experiments offer the advantage of faster process development due to decreased time of steady inoculum preparation and separation of biomass growth from physiology manipulation experiments.

Another improvement of the classical chemostat is the accelerostat cultivation method where the dilution rate of culture (defines μ in steady state) is changed slowly enough for the cells to remain in a state comparable with steady state [65]. This system has the benefit of reduced experimental time as there is no need to stabilize culture for each new μ and has been shown to be highly similar to chemostat cultivations [5]. The accelerostat method has been mainly used for microbial physiology studies for the determination of metabolic rearrangements with changing μ (reviewed in [5]). Meier et al. applied the accelerostat technique in the process development of a monoclonal antibody production in *Hansenula polymorpha* and demonstrated highest space-time-yield near maximum μ of the organism [66]. Recently, the accelerostat technique was used to determine the optimal μ for endo-polygalacturonase production in *S. cerevisiae*. In the continuous culture, two optima were found, one at a lower μ below the critical specific growth rate for aerobic overflow metabolism of ethanol, and the other at a higher μ. Interestingly, in fed-batch cultivations, only the lower optimal μ was confirmed while the q_p_ was low at higher μ. [67]

## 5. Steady-State Bioprocess Optimization in Continuous Cultures

Besides μ-dependent product formation kinetics studies, continuous cultures have been used to optimize many other process parameters. We summarized selected examples that demonstrate the versatile uses of continuous cultures in recombinant process development in Table 2.

Controlled and constant environment provided by continuous cultures is an advantage that can be effectively used to study the effect of growth conditions on plasmid stability. Brigidi et al. compared several plasmids for α-amylase production in *Bacillus stearothermophilus* and identified a system that could be maintained for more than 300 generations without significant plasmid loss [68]. Others have used continuous cultures to screen optimal growth conditions for plasmid maintenance in non-selective medium and auxotrophic complementation systems [57,69,70]. Partow et al. used the steady-state environment for a detailed comparison of plasmid-based promoter strengths and created a novel expression system for use in glucose-based media [71].

Metabolic flux analysis has been widely used for identification of major intracellular pathways and critical branch points in metabolic networks. Isotope incorporation analysis is considerably simplified in steady-state conditions and therefore is most often carried out in chemostats. Fürch et al. studied the metabolic link between glycolysis and the tricarboxylic acid (TCA) cycle in *Bacillus megaterium* using ^13^C-labelled glucose and pyruvate [72]. They showed that a significant improvement in recombinant hydrolase production could be achieved using pyruvate. Increase in both the adenosine triphosphate (ATP) and nicotinamide adenine dinucleotide (NADPH) yields were determined on pyruvate, providing a more optimal supply of both precursors as well as energy and reduction equivalents for recombinant protein expression [72]. Pfeffer et al. developed a novel ^34^S labelling procedure for the flux measurements of a recombinant protein producing *P. pastoris* [73]. Using carbon-limited continuous cultures, they discovered that from the total amount of protein produced intracellularly, about 58% are degraded within the cell and only about 35% are secreted [73].

With the use of GAP promoters, often a strong positive correlation between q_p_ and µ have been reported (Table 1). Buchetics et al. used continuous cultures to analyze the correlation of cell cycle and protein secretion rate of GAP-dependent expression of Fab in *P. pastoris* [48]. They showed that q_p_ was more correlated with cell cycle than µ, and based on the results, constructed strains with customized cell cycle phase distribution. Resulting strains had increased q_p_ at low µ, and increased space-time-yield of the target proteins Fab and trypsinogen [48].

Besides the more advanced specific cases described above, there are many other process parameters that have been optimized using steady-state continuous cultures: feed composition [74], temperature [75,76], pH [52,55,77], dissolved oxygen [52], and inducer concentration [62]. Although these process parameters can also be optimized in batch and fed-batch cultures, using continuous cultures will avoid secondary effects arising from changing µ or biomass density.

## 6. Continuous Manufacturing of Recombinant Proteins

Growing cells in continuous cultures results in a steady-state operation, which is easier to characterize and understand, but it can also have economic benefits. Many growing industries have at one point made a switch from batch to continuous manufacturing and this has had a tremendous effect on their efficiencies [9]. Several examples of continuous bioprocesses can be found, including wastewater treatment, composting, biogas, ethanol, and single cell protein production [78,79]. Maintaining cells in a producing state for extended time periods is a major advantage that can lead to high volumetric productivities in continuous bioprocesses. In case of processes with microorganisms, the space-time-yields can be especially high due to their ability to grow very fast. Several other benefits, such as lower setup and running costs, reduced equipment size, product quality and process scalability, have been associated with continuous bioprocesses [8,9]. Continuous manufacturing in smaller production vessels could also decrease the problems associated with process scale-up as pilot- and production-scale processes can be carried out using more similar equipment. Even though high efficiencies of continuous recombinant protein processes with different microorganisms can be found in the scientific literature [39,63,80], their commercialization is limited. As adaptation is driven by economics, the risks associated with continuous processes must be considered. Several issues possibly affecting continuous manufacturing have been raised: challenges with long-term stability and sterility, poor short-term flexibility due to long run times and genetic instability of cells to name a few [81]. Some attempts have been made to create lower mutation rate hosts [82], but it is difficult to eliminate this risk completely. Novel selection marker strategies [57], tunable expression [7], and bioprocess strategies [69] have been used to mitigate the stability issues. Even if most of the hurdles can be overcome, the choice between batch and continuous process finally depends on the longer-term objectives of the facilities as large rearrangements or totally new systems are required. Possibly for these reasons, examples of continuous recombinant protein productions are exclusively for the manufacturing of high demand blockbuster biopharmaceuticals, for which semi-continuous perfusion technologies and mammalian cell cultures are used [83]. The only known example of continuous industrial recombinant process applying microorganisms dates back to the 1990s, when insulin was produced with *S. cerevisiae*, but also in this case, process stability was problematic [84].

Besides the cultivation side, a production facility must also consider downstream operations that need to be coupled with the upstream operations to maximize the benefits of a truly continuous bioprocess. There are many recent developments in the direction of fully end-to-end continuous recombinant protein production processes, which include downstream purification, cell lysis, and protein refolding [85,86]. More recently, Kateja et al. developed an end-to-end integrated continuous downstream process for a therapeutic protein expressed in *E. coli* as inclusion bodies [6]. Furthermore, Klutz et al. proposed a complete biofacility design based on a fully continuous processing and single-use technology [87]. A recent patent on the integration of cultivation and downstream chromatographic systems also demonstrates the growing industry interest in fully continuous recombinant protein production processes [88].

## 7. Conclusions

Within this contribution, we focus on the application of continuous cultures for different aspects of microbial recombinant protein production. Furthermore, we describe how methods of keeping cells in constant environment using continuous cultures have been used to advance basic understanding of recombinant cell physiology and for process development in microorganisms.

## Figures and Tables

**Table 1:** µ-dependent product formation kinetics in continuous cultures. Abbreviations used: IFN: interferon; scFv: Single-Chain Variable Fragment Antibody; GM-CSF: granulocyte- macrophage colony stimulating factor; VHH: variable domain of heavy chain antibody.

Organism	Promoter	Target Protein	µ-Range (h^−1^)	Optimal µ (q_p,max_) (h^−1^)	q_p,max_ (mg g^−1^ h^−1^) or (U mg^−1^ h^−1^)	Kinetics	Ref
*E. coli*	*Pbla*	β-lactamase	0.23–0.64	0.44	3.6 (U mg^−1^ h^−1^)	bell shaped	[34]
*E. coli*	*P_L_/cI*_857_	Lymphokine	0.027–0.25	0.17	6.5	bell shaped	[35]
*E. coli*	*Pbla*	β-lactamase	0.14–1	0.49	11 (U mg^−1^ h^−1^)	bell shaped	[36]
*E. coli*	*Plac*	Cyanase	0.05–0.93	0.15	8 (U mg^−1^ h^−1^)	bell shaped	[37]
*E. coli*	*T7*	IFN-α	0.1–0.5	0.37	170	growth coupled	[32]
*E. coli*	*T7*	IFN-γ	0.1–0.3	0.3	75.00	growth coupled	[33]
*E. coli*	*CP7*	β-glucanase	0.1–0.5	0.15	14 (U mg^−1^ h^−1^)	bell shaped	[57]
*P. pastoris*	*AOX*	scFv	0.009–0.05	0.02	0.12	bell shaped	[40]
*P. pastoris* ^a^	*AOX*	scFv	0.007–0.05	0.04	0.004	linear	[40]
*P. pastoris*	*AOX*	Trypsinogen	0.03–0.2	0.07	0.69	bell shaped	[39]
*P. pastoris*	*AOX*	Chymotrypsinogen B	0.038–0.078	0.078	0.38	growth coupled	[42]
*P. pastoris*	*AOX*	Antifreeze protein	0.01–0.09	0.09	0.065	growth coupled	[43]
*P. pastoris*	*AOX*	Avidin	0.03–0.12	0.12	0.027	growth coupled	[41]
*P. pastoris*	*GAP*	Serum Albumin	0.015–0.15	0.15	0.174	growth coupled	[27]
*P. pastoris*	*GAP*	Fab	0.02–0.19	0.19	0.049	growth coupled	[47]
*P. pastoris*	*GAP*	Fab	0.02–0.19	0.19	0.047	growth coupled	[48]
*P. pastoris*	*GAP*	Fab	0.025–0.15	0.15	0.047	growth coupled	[58]
*P. pastoris*	*GAP*	GM-CSF	0.02–0.2	0.2	0.5	growth coupled	[49]
*P. pastoris*	*THI11*	Serum albumin	0.05–0.15	0.15	0.18	growth coupled	[50]
*H. polymorpha*	*MOX*	α-galactosidase	0.05–0.2	0.08 and 0.18	5.5	2 optima	[45]
*S. cerevisiae*	*GAL7*	α-galactosidase	0.05–0.225	0.17	4.5	bell shaped	[45]
*S. cerevisiae*	*GAL7*	VHH	0.033–0.172	0.172	3.87	growth coupled	[44]
*S. cerevisiae* ^b^	*GAL7*	VHH	0.033–0.147	0.09	1.7	bell shaped	[44]
*S. cerevisiae*	*GAL7*	Cutinase	0.05–0.1	0.07	8	bell shaped	[46]
*S. cerevisiae*	*TPI1*	α-amylase	0.5–0.2	0.2	55 (µmoL mg^−1^ h^−1^)	growth coupled	[51]
*S. cerevisiae*	*TPI1*	Insulin precursor	0.5–0.2	0.2	12 (µmoL mg^−1^ h^−1^)	growth coupled	[51]
*K. lactis*	*PGK*	Serum albumin	0.05–0.19	0.19	0.8	growth coupled	[52]
*K. lactis* ^c^	*PGK*	Serum albumin	0.05–0.12	0.12	0.225	growth coupled	[52]
*A. niger*	*PglaA* ^d^	Glycoamylase	0.06–0.14	0.14	12	growth coupled	[53]
*A. niger*	*PglaA*	Glycoamylase	0.05–0.13	0.1	7	growth coupled	[54]
*A. niger*	*PglaA* ^d^	Glucoamylase	0.05–0.26	0.23	15	growth coupled	[55]
*A. niger*	*Native* ^e^	Acid phosphatases	0.04–0.13	0.13	7.5 (kU mg^−1^ h^−1^)	growth coupled	[56]

^a^ Constant limited dissolved oxygen at 10%; ^b^ Nitrogen limited cultures; ^c^ High cell density culture with 60 g L^−1^ glucose; ^d^ 20 copies of *glaA* gene with its native chromosomal promoter; ^e^ Several acid phosphatases expressed from their native promoters.

**Table 2:** Main results of selected recombinant processes optimized in continuous cultures.

Organism	Target Protein	Optimisation Criteria	Main Results	Ref
*B. stearothermophilus*	α-amylase	Plasmid stability	Several stable plasmids identified (up to 200 generations tested).	[68]
*S. cerevisiae*	β-Galactosidase	Promoter strength characterisation	Novel expression vectors designed with strong constitutive expression for use in glucose media.	[71]
*B. megaterium*	Hydrolase	Metabolic flux distribution in the pyruvate node	Optimal metabolic flux distribution in pyruvate-based medium led to increased q_p_.	[72]
*P. pastoris*	Fab	Protein secretion and degradation quantification in vivo	^34^S labelling identified 58% degradation and 35% secretion of Fab.	[73]
*P. pastoris*	Fab and trypsinogen	μ-Dependent protein secretion re-wiring	Strain created with increased protein secretion at low μ, improved space-time-yield.	[48]
*P. pastoris*	β-galactosidase	Methanol: sorbitol co-feeding	0.45–0.75 C-moL/C-moL of methanol fraction optimal for pAOX induction; reduced oxygen demand.	[74]
*E. coli*	Fab	Optimal temperature	30 °C favored over 33 °C and 37 °C for Fab production without detriment to biomass yield.	[76]
*K. lactis*	Serum albumin	Optimal pH (tested range: 4–8)	Highest q_p_ reached at pH 6.5.	[52]
*K. lactis*	Serum albumin	Optimal dissolved oxygen (tested range: 5–80%)	Highest q_p_ reached at lowest dissolved oxygen conditions (5%) tested.	[52]
*E. coli*	mCherry	Induction strategy	Dynamic increase of the inducer led to an increase in the product concentration of 21%	[62]
